# Global Sensitivity Analysis for Robust XAI: Quantifying Clinical Risk and Prediction Instability in Dermoscopic Image Classification

**DOI:** 10.1111/risa.70237

**Published:** 2026-03-30

**Authors:** Giulia Vannucci, Renato Patrik Williame Coppolecchia, Roberta Siciliano

**Affiliations:** ^1^ Department of Electrical Engineering and Information Technology Polytechnic and Basic Sciences School, University of Naples Federico II Napoli Italy; ^2^ Department of Physics University of Naples Federico II Napoli Italy

**Keywords:** clinical risk, convolutional neural network, dermoscopic images, global sensitivity analysis

## Abstract

The high nominal accuracy achieved by deep learning models in predicting malignant skin lesions is frequently undermined by their susceptibility to operational uncertainty. Image acquisition conditions, such as lighting, device settings, and skin characteristics, introduce variations in optical parameters that compromise the model's reliability in real‐world clinical settings. This instability produces an unquantified diagnostic risk, which makes the safe clinical implementation of these powerful systems difficult. With this critical gap in mind, this paper proposes and utilizes global sensitivity analysis to rigorously quantify the robustness of a convolutional neural network architecture with respect to five critical optical image parameters. The analysis aims to quantify the model's instability, moving beyond simple accuracy to provide a robust, risk‐quantified assessment. This approach is essential for establishing the level of confidence required for the accreditation and safe clinical deployment of AI‐based diagnostic systems.

## 1 Introduction

Melanoma is one of the main types of skin cancer and is the most dangerous form due to its high potential for metastasis, capable of spreading to regional lymph nodes and distant organs such as the lungs, liver, bones, and brain. An effective diagnosis is crucial in terms of timing: several studies indicate that, compared to patients treated within 30 days after biopsy, those treated 30–59 days later face a 5% higher risk of death. This risk can increase further by 41% if treatment is delayed beyond 119 days (source: www.skincancer.org, see, e.g., Strazda et al. 2024, for a systematic review and meta‐analysis). Thus, a rapid diagnosis is directly related to a better patient's prognosis. The primary risk factors for melanoma include exposure to UVA and UVB rays—UVA being the most abundant and penetrating and UVB responsible for sunburn—with both classified as carcinogens of Group 1 (Fadadu and Wei 2022). The traditional diagnostic process, based on a clinical approach, achieves good classification rates, but often requires additional histological examinations that involve long waiting times. The inherent subjectivity of this process, combined with delays, poses a significant challenge to public health.

Recent advances in skin disease identification have witnessed a surge in the use of deep learning (DL) techniques, particularly convolutional neural networks (CNNs) (Gulshan et al. 2016). These architectures have demonstrated remarkable performance in various dermatological applications, including skin lesion classification and semantic segmentation (Ronneberger et al. 2015). Integration of AI allows for a data‐driven diagnosis approach, promising classification accuracy exceeding 90% and offering the potential for real‐time response, thus reducing or eliminating the need for prolonged waiting times associated with histological examinations (Maron et al. 2020).

However, despite the high nominal performance in controlled environments, the transition of DL models to actual clinical practice necessarily faces the challenge of operational uncertainty. Image acquisition conditions, such as lighting, the device used, and skin characteristics, are highly variable factors (Li et al. 2023). Inherently, they introduce variations in image characteristics that can greatly affect the reliability of the model used. CNNs are notoriously “black‐box” systems, in which the transformation of input images into predictions remains opaque, making it difficult to interpret how specific outputs are generated. In the context of clinical risk quantification, the lack of transparency and robustness directly translates into an unquantified diagnostic risk, hindering clinical confidence in the implementation of such techniques and limiting the potential use of the model (Fehr et al. 2024).

Here, to move beyond simple accuracy metrics, we adopt the global sensitivity analysis (GSA) methodology to quantify the contribution of uncertain input factors of image acquisition to the total variance of a model's output (Saltelli et al. 2008). By systematically exploring the entire domain of input factor uncertainty through quasi‐random sampling, GSA provides a crucial, data‐driven foundation for risk assessment in highly variable clinical environments, moving the focus from nominal performance to quantifiable robustness. GSA is an established methodology applied in various domains. A brief history of the discipline can be found in Tarantola et al. (2024). While recent comprehensive reviews of the state‐of‐the‐art can be found in Saltelli et al. (2021), the methodology has proven particularly effective in characterizing complex “black‐box” models (Iooss and Lemaître 2015) and biological systems (Marino et al. 2008). Furthermore, the importance of sensitivity measures in the context of model robustness and reliability is increasingly recognized in the machine learning community (Borgonovo and Plischke 2016; Razavi et al. 2021). Other examples of the GSA approach can be found in Becker et al. (2021), where it is used for variable selection in regression settings, while in Vannucci et al. (2024) it is employed as a novel approach to understanding variable importance in Random Forests (Breiman 2001).

In this paper, we use the GSA approach to explore the operational domain of uncertainty associated with photometric perturbations of dermoscopic images and to quantify how the variance in input parameters is reflected in the variance of the model output. The objective is to identify which factors and interactions account for the largest proportions of variance in the predicted probability of melanoma. We adopt an explicit operational notion of risk tailored to AI‐based diagnostic systems. Risk is defined here as the instability of the diagnostic output under plausible operational uncertainty, rather than as a clinical loss or error rate. Uncertainty is represented through distributions over image acquisition parameters, while severity is reflected in the magnitude of changes in the predicted probability of melanoma, particularly when such changes approach or cross decision‐relevant thresholds. Within this framework, GSA is used to identify the dominant drivers of diagnostic risk, defined as output variance under a specific perturbation model, as an alternative to providing case‐level mechanistic explanations. We thus frame this approach as Robust explainable AI (XAI). While traditional XAI methods aim to explain individual predictions, Robust XAI employs global sensitivity measures to provide a diagnostic of model stability. In this context, Sobol indices serve as importance measures that quantify variable importance under operational uncertainty, identifying which factors contribute most to prediction instability across a broad range of plausible scenarios.

The remainder of the paper is structured as follows. Section 2 presents the real‐world dataset and the challenging goals. Section 3 provides the workflow of the proposed explainable AI (XAI) methodology. Section 4 describes the DL models with the selected architectures. Section 5 provides the variance‐based GSA and the setup for a robust assessment of the dermatoscopic image classification. Section 6 provides the results of the robust XAI methodology. Finally, Section 7 provides concluding remarks and future research directions.

## 2 Human Against Machine Dataset and Goals

**FIGURE 1:**
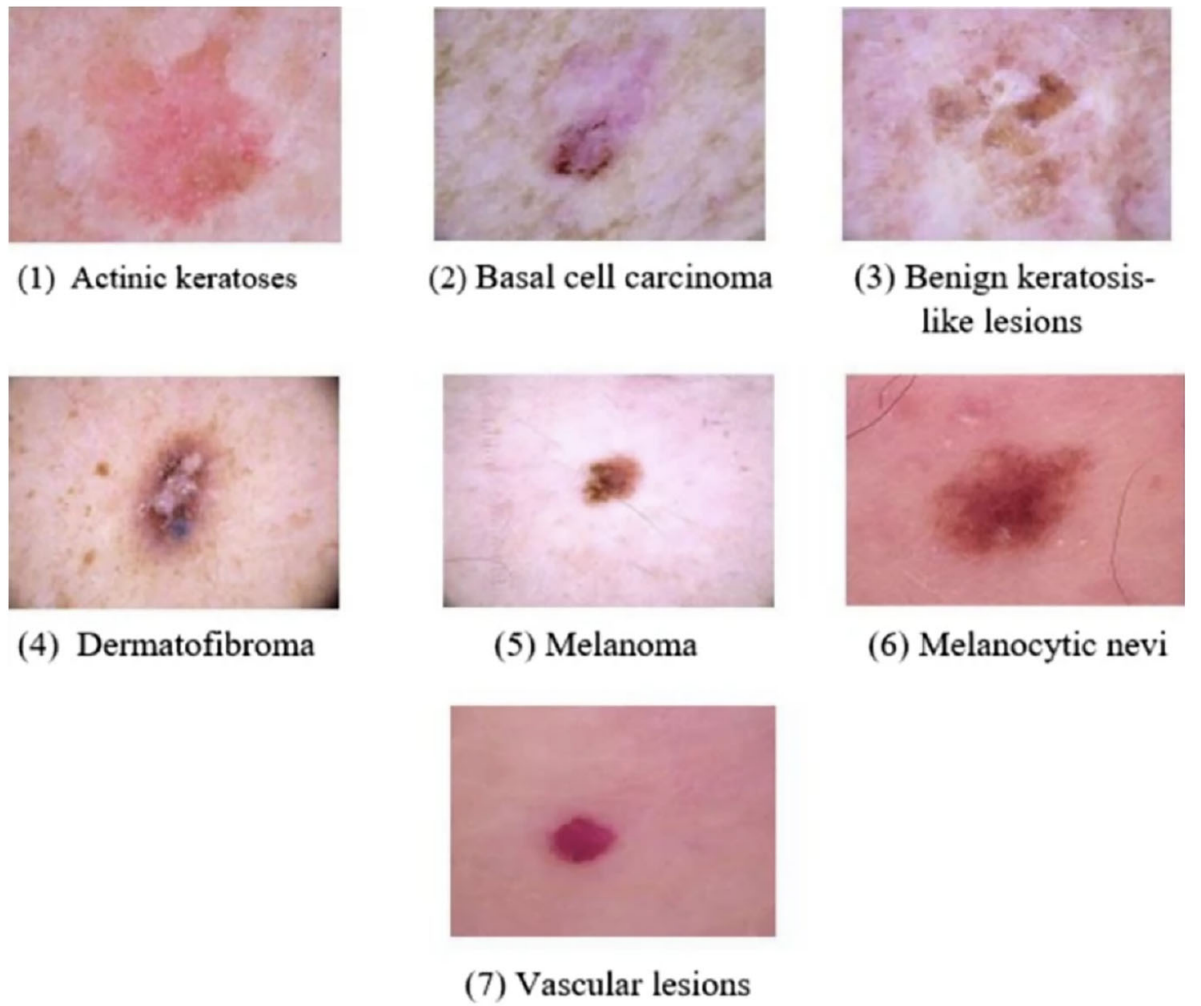
Example images illustrating various types of skin lesions present in the HAM10000 dataset.

We utilized the Human Against Machine (HAM10000) dataset, a publicly available collection of 10,015 dermoscopic images (Tschandl et al. 2018) for the development and evaluation of AI systems in the diagnosis of pigmented skin lesions. Contributions were sourced from the Medical University of Vienna, Austria, and Cliff Rosendahl, Queensland, Australia. All images were fully anonymized and sourced with appropriate ethical approval from the original providing institutions. The dataset encompasses seven distinct diagnostic classes, with several diagnoses consolidated for simplicity: melanocytic nevi, melanoma, benign keratosis‐like lesions (solar lentigines/seborrheic keratoses), basal cell carcinoma, actinic keratoses and intraepithelial carcinoma, vascular lesions, and dermatofibroma. Figure 1 shows the different types of skin lesions available in the dataset. Each image is accompanied by detailed patient metadata, including lesion_id, image_id, dx (diagnosis), dx_type (verification method), age, sex, and localization (body site of the lesion). The lesions were acquired using various modalities, including dermatoscopes, digital cameras, and smartphones. More than half of the lesions have been histopathologically confirmed, with the remaining verified through expert consensus, follow‐up examinations, or in vivo confocal microscopy (Tschandl et al. 2018).

**FIGURE 2:**
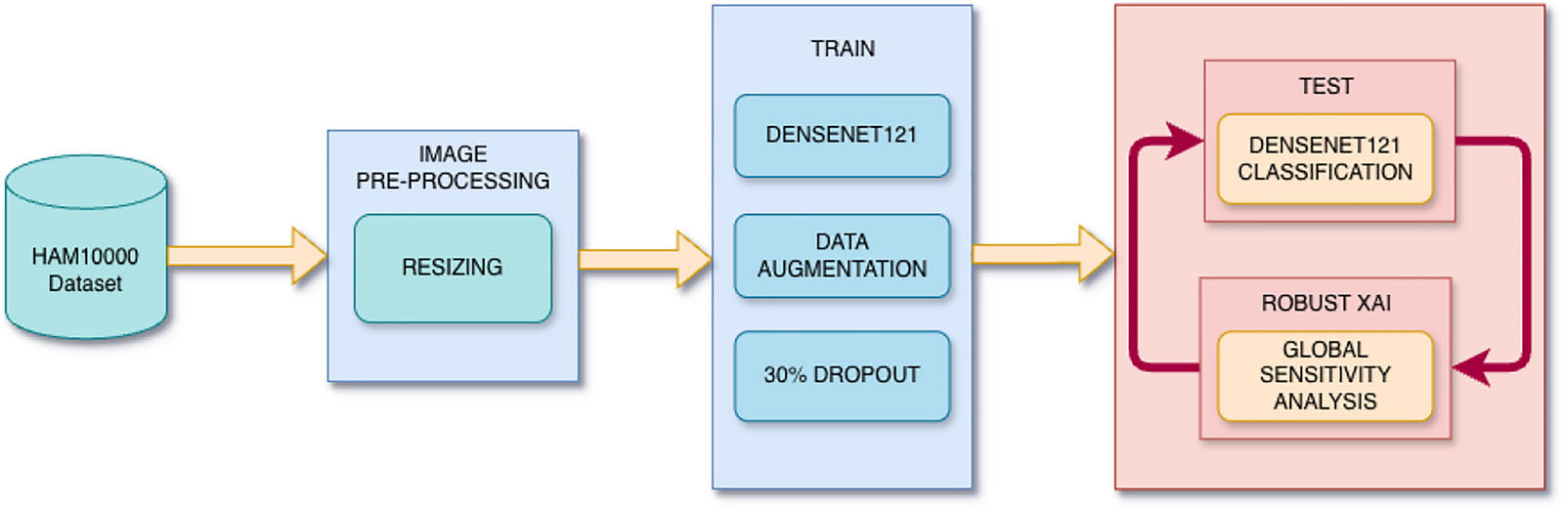
Robust XAI methodological workflow.

**FIGURE 3:**
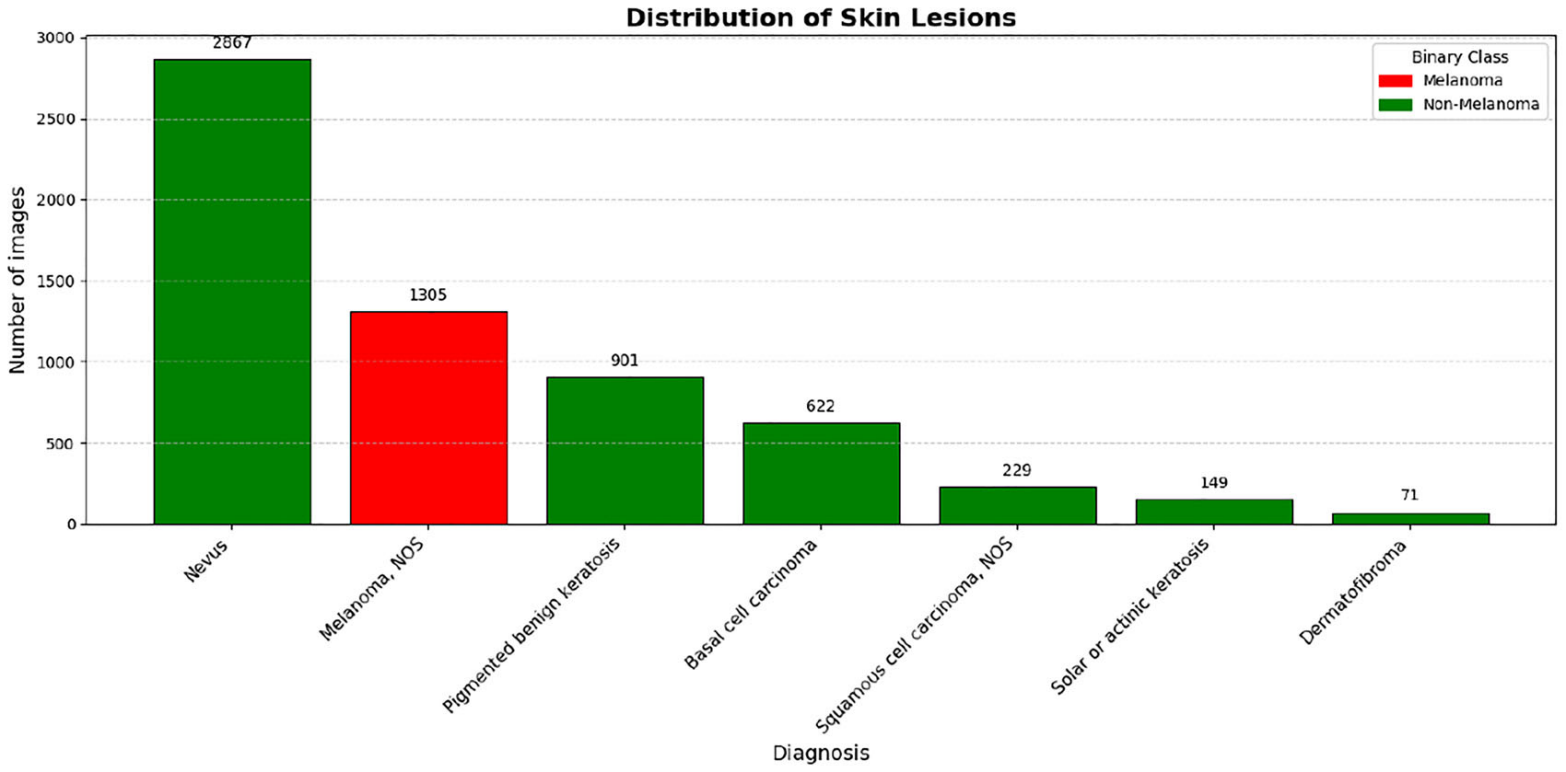
Distribution of skin lesions in the HAM1000 dataset filtered by confirmed histological diagnosis.

Initial analysis of the HAM10000 dataset revealed critical characteristics regarding the distribution of classes and demographics of the patients (see Figure 3), which require specific considerations during model training. The only malignant type of skin lesion present in this dataset is malignant melanoma, later called melanoma, where surgical removal in the early stage of cancer can provide a cure. The remaining skin lesions are benign, although they may require treatment, in particular basal cell carcinoma, which is a local skin cancer, and actinic keratoses, which can transform into melanoma.

## 3 Robust XAI Methodological Workflow

The methodological core of this study is based on a data science strategy designed to bridge the gap between the high performance of DL models and clinical interpretability. We want to enrich a “black‐box” model that is built with the goal of accuracy in performance, with GSA aiming to quantify the operational uncertainty of the model, in order to make it both robust and interpretable. As illustrated in the methodological workflow in Figure 2, the process was structured starting from the HAM10000 dataset, with a preprocessing phase in which the images are first subjected to histological filtering and uniform resizing. Then, the DenseNet‐121 architecture was used, incorporating both data augmentation (DA and a dropout rate 30% to improve generalization and mitigate overfitting. Finally, to go beyond standard performance evaluation, we used GSA to partition the model output variance across a space of optical perturbations of the images. This allows us to characterize the diagnostic risk associated with the variability of image acquisition in the real world. In this context, we use the term “Robust XAI” to denote a complementary perspective to traditional explainable AI methods. Most XAI approaches aim to provide local explanations for individual predictions, whereas our framework employs GSA to assess the stability of model outputs under operational uncertainty. Therefore, Sobol indices should be interpreted as global variance‐attribution measures under the specified perturbation model, rather than as mechanistic explanations of individual predictions.

In the following sections, a detailed description of each stage of this workflow is provided, outlining the key methodological choices and specific configurations adopted in this study.

## 4 Image Analysis and Deep Neural Network

### 4.1 CNN Architecture

The transition from a raw dermoscopic image to a definitive diagnosis requires a structured image analysis processing, aimed at image conditioning, lesion isolation, and salient feature quantification for algorithmic analysis. Three steps comprise the image analysis processing: the image preprocessing, the image segmentation, the extraction of features, and classification. Image preprocessing techniques are used to understand the image content in a better way so that analysis can be done effectively. The goal is to identify the interest points in the image, which helps in feature extraction. Preprocessing performs operations with raw images at the pixel level, where both input and output are intensity images. The main object of this phase is to improve the image data by removing annoying distortions and to enhance the significant image features for further processing (Sonka et al. 1993). The main preprocessing techniques are based on the application of various image filters, which modify pixel values based on specific mathematical kernels. In dermatoscopic applications, nonlinear filters such as the median filter (Lee 1990; Abbas et al. 2011) or gradient‐based operators such as the Sobel filter or Canny edge detector (Gonzalez 2009; Abdolrazzaghi et al. 2022) are mainly used.

Image segmentation partitions the digital image into meaningful subgroups to isolate the lesion from the surrounding skin. Although our final classification utilizes an end‐to‐end approach, it is noted that DL methods are highly effective for pixel‐level segmentation, with state‐of‐the‐art architectures in this domain, including U‐Net (Ronneberger et al. 2015), known for its skip connections, DeepLab (Chen et al. 2018), using atrous convolution, and Mask R‐CNN (He et al. 2017) for instance segmentation. Feature extraction and classification activities can be performed using deep neural networks (DNN). DNNs are layered architectures of interconnected computational units (neurons), conceptually inspired by biological neural systems to emulate the brain's ability to recognize complex patterns. The main calculation is the weighted sum of the inputs ($\text{net}=\sum_{i=1}^{n}W_{i}X_{i}$) that go through a nonlinear activation function (f). Even if standard neural networks (NNs) are capable of processing extracted feature vectors. CNNs represent a transformative advancement that automatically learns complex, descriptive, and hierarchical features directly from raw images, learning from pixel intensities. This end‐to‐end feature learning helps eliminate the need for manual feature engineering.

A typical CNN architecture consists of a sequence of functional layers: convolutional, activation, pooling, batch normalization, and dropout, culminating in fully connected layers. The network handles feature extraction in the initial layers, while the subsequent layers stabilize the training process and produce the final classification output.

The high hierarchical structure as well as the vast learning capacity of DNNs makes the CNN architecture an effective backbone for classification tasks. The Xception architecture (Chollet 2017) is a very efficient CNN that uses separable convolutions in depth to capture features at different scales. It is based on an “extreme inception” model. This method breaks the standard convolution down into two useful steps: depthwise and pointwise convolution. This makes it much easier to use, as it cuts down on the number of parameters compared to traditional CNNs. Xception has a linear architecture with residual connections that are very important to solve the vanishing gradient problem and to allow training very deep networks. DenseNet (Huang et al. 2017) is a densely connected convolutional network that forwards connects each layer to the other layers. It is characterized by its dense connectivity pattern, which directly connects the feature maps of all preceding layers to every subsequent layer. The key relationship is that the feature maps of all previous layers $X_{0},X_{1},\text{…},X_{l-1}$ are combined and used as input for layer $X_{l}$. As a result, the l‐layer $X_{l}$ receives the feature maps of all preceding layers as input. Therefore, $X_{l}=H_{l}\left(\left[X_{0},X_{1},\text{…},X_{l-1}\right]\right)$, where [$X_{0},X_{1},\text{…},X_{l-1}$] indicates the integration of the feature maps produced in layers 0,…,l−1, and the multiple inputs of $H_{l}\left(\cdot \right)$ are concatenated into a single tensor. This architecture provides several advantages: mitigation of vanishing gradient, increased feature reuse, and improved parameter efficiency. Variants such as DenseNet‐121 and DenseNet‐201 differ primarily in depth and complexity: DenseNet‐121 comprises 121 layers and represents a highly efficient configuration, widely adopted for image classification tasks.

### 4.2 Data Preprocessing and DenseNet Architecture

Given that standard CNNs typically require fixed‐size input tensors, the image preprocessing step involved the uniform resizing of all the images. Based on computational constraints and common practices in DL for dermoscopy, a dimension of 128×128 pixels was selected. This choice balances the need to retain sufficient visual information with the need to manage memory constraints during training. To suppress common dermoscopic artifacts, such as hair, an automated process was implemented using standard image processing techniques (Gonzalez 2009). However, subsequent classification experiments revealed that these algorithms did not yield improved results, likely because the filtering methods inadvertently removed or blurred subtle lesion features critical for classification. Consequently, these “cleaned” images were not used for the final classification task.

We decided to focus our analysis on the identification of the malignancy in skin disease, which represents the primary clinical objective. Crucially, to ensure the highest clinical reliability of the labels, the dataset was filtered to include only lesions with biopsy confirmation. This reduced the working set from the original HAM10000 collection to a final total of 6,227 images for our analysis. Then, the initial seven‐class diagnostic outcome was dichotomized into two mutually exclusive groups, resulting in a new variable denoted as melanoma, where the malignant class is encoded as 1 (melanoma) and the remaining lesions are grouped under 0 (non‐melanoma). Due to the inherent class imbalance, the filtered dataset was then divided into training, validation, and test subsets using stratified sampling to maintain a consistent representation of the melanoma class across all splits. The final distribution of images across the three sets is 4358 in the training set, 934 in the validation set, and 935 in the test set. Then, we adopt the DA technique (Mikołajczyk and Grochowski 2018). It is an essential regularization implemented “on‐the‐fly.” The DA pipeline included specific geometric and photometric transformations to increase the dataset variety, consistent with the final training configuration:

- Flipping: Random horizontal and vertical flipping.
- Rotation: Random rotation up to 0.2 radians (≈±11.5 degrees).
- Zoom: Random scaling up to 20% (randomly zooming in or out).
- Contrast: Random contrast adjustment up to 20%.

The core of the classification system is based on the DenseNet‐121 architecture, which was selected for its high parameter efficiency and its ability to mitigate the vanishing gradient problem through its characteristic dense connectivity pattern (see Figure 3). To ensure that the correct normalization is applied consistently, particularly during the GSA perturbation process, the image preprocessing layer was integrated directly into the model's structure. The input tensor of the 128×128×3 pixels is first processed by a Lambda layer that applies the DenseNet‐specific normalization. To guarantee that the GSA captures the model's response to the raw pixel values before normalization, the DenseNet‐121 architecture was modified to include the preprocessing stage (mean subtraction and variance scaling) as an internal Lambda layer. Consequently, the input vector X for the Sobol analysis consists of raw pixel intensities in the [0,255] range. This architectural choice ensures that the resulting sensitivity indices represent a formal attribution of output variance to operational input factors. By using Sobol indices, we provide a global robustness diagnostic that quantifies variable importance under uncertainty, ensuring that our results reflect the statistical sensitivity of the entire diagnostic pipeline from image acquisition to final prediction. The model was initialized using weights pretrained on the ImageNet dataset, employing a standard transfer learning approach. The DenseNet‐121 base model was extended with a custom classification head tailored for binary output: the feature maps of the base model were aggregated using a combination of global average pooling and global max pooling, which were then concatenated. This strategy aims to capture both general features and the most salient features of the final convolutional block. Then, the concatenated feature vector was fed into a fully connected layer (Dense(256), followed by a dropout layer with a rate of 0.3), and culminated in the output layer, consisting of a single unit with a sigmoid activation function to predict the probability of melanoma.

The training process utilized a robust two‐phase strategy to maximize both initial convergence speed and final fine‐tuning performance. In Phase 1, the weights of the DenseNet‐121 base model were frozen and only the parameters of the custom classification head were trained. This phase focused on quickly adapting the new layers to the melanoma classification task, using the Adam optimizer with an initial learning rate of 0.001 and minimizing the binary cross‐entropy loss function. Following the initial adaptation, the model underwent Phase 2, a procedure to subtly adjust the feature extraction layers of the base model. This involved selective unfreezing of the last 20% of the DenseNet‐121 convolutional layers, while the initial layers remained frozen to preserve the benefit of the ImageNet pretraining. The model was recompiled and trained with a significantly reduced learning rate of 1e−5 to prevent large gradient updates from corrupting the pretrained weights. Throughout both training phases, optimization was meticulously guided by clinical relevance and stability. Key diagnostic metrics such as area under the curve (AUC), recall, and precision were monitored along with accuracy. Furthermore, the training process used a control mechanism via callbacks that monitored the AUC in the validation set. Specifically, early stopping with patience of 15 epochs was utilized to prevent overfitting and identify the optimal training iteration, and the reduced learning rate with patience of five epochs was implemented to dynamically adjust the learning rate, ensuring stable and robust convergence by reducing the step size upon stagnation of the performance metric.

## 5 Global Sensitivity Analysis for Robustness Assessment

### 5.1 The Method

The complexity of quantitative models in critical applications, such as medical image analysis, requires systematic robustness and sensitivity analyses to handle inherent uncertainties (Chiani et al. 2025). An established methodology capable of quantifying how uncertainty in a model's output can be attributed to the different sources of uncertainty in its inputs is GSA (Saltelli et al. 2008). Unlike local sensitivity methods, GSA explores the entire domain of input factor uncertainty, providing a crucial advantage for risk assessment in a highly variable operational environment. The model is treated as a generic function Y=f(X), where Y is the predicted probability of melanoma (our quantity of interest), and X is the vector of uncertain image perturbation factors.

The most effective technique for GSA in complex, nonlinear models is the variance‐based sensitivity analysis (VB‐SA), popularized by the work of Sobol' and Saltelli. VB‐SA allows input factors to be ranked according to their contribution to the output variance V(Y). Crucially, VB‐SA also tackles interaction effects instructing the analyst about cooperative behavior of factors; interactions can lead to extreme values of model output and are thus highly relevant to stability analysis. In VB‐SA, the two most relevant measures are the “first‐order” and “total order” indices.

The best systematization of the theory of variance‐based methods is due to Sobol' (Sobol' 1990), while total sensitivity indices were introduced by Homma and Saltelli (1996). For reviews, see also Saltelli et al. (2005), Helton et al. (2006), or Vannucci et al. (2025). VB‐SA uses measures such as the first‐order Sobol index ($S_{i}$), that is, the fraction of the total variance V(Y) attributed to the factor $X_{i}$ alone:

(1) $$S_{i}=\frac{V_{X_{i}}\left(E_{X_{∼i}}\left(Y|X_{i}\right)\right)}{V(Y)}$$

and the total Sobol index ($S_{T_{i}}$), that is, the fraction of V(Y) attributed to the factor $X_{i}$ and all its interactions with the other factors:

(2) $$S_{T_{i}}=\frac{E_{X_{∼i}}\left(V_{X_{i}}\left(Y|X_{∼i}\right)\right)}{V(Y)}=1-\frac{V_{X_{∼i}}\left(E_{X_{i}}\left(Y|X_{∼i}\right)\right)}{V(Y)},$$

where $X_{∼i}=\left\{X_{1},X_{2},\text{…},X_{i-1},X_{i+1},\text{…},X_{k}\right\}$ denotes the set of all input factors except $X_{i}$. The term $E_{X_{∼i}}\left(Y|X_{i}\right)$ is the value of Y obtained by averaging over all factors except $X_{i}$ and is thus a function of $X_{i}$ alone. $V_{X_{i}}\left(E_{X_{∼i}}\left(Y|X_{i}\right)\right)$ is the variance of this function over $X_{i}$ itself. Intuitively, a high value of these statistics implies an influential factor. The quantity $S_{i}$ corresponds to the fraction of V(Y) that can be attributed to $X_{i}$ alone. It can be viewed as a measure of how well $E_{X_{∼i}}\left(Y|X_{i}\right)$ fits Y: if the fitting is optimal, then $S_{i}≅1$ and the factor $X_{i}$ is highly relevant. The quantity $S_{T_{i}}$ corresponds to the fraction of V(Y) that can be attributed to $X_{i}$ and all its interactions with other factors. For additive models, the two measures $S_{i}$ and $S_{T_{i}}$ are equal to one another for each factor $X_{i}$. For an interacting factor, the difference $S_{T_{i}}-S_{i}$ is a measure of the strength of the interactions (Saltelli et al. 2008).

Estimation of $S_{i}$ and $S_{T_{i}}$ requires the computation of k‐dimensional integrals. They are generally approximated by assuming independency among input factors and using Monte Carlo or quasi Monte Carlo sampling from the joint distribution of the space of input factors. Alternative procedures for the computation of $S_{i}$ and $S_{T_{i}}$ that use direct calculations are available. They all derive from metamodels, which provide cheap emulators of complex and large computational models (see, e.g., Oakley and O'Hagan 2004; Storlie et al. 2009).

### 5.2 Parameter Uncertainty Bounds

We conducted a GSA to evaluate the robustness of the proposed model with respect to variations in image preprocessing. Five key factors were analyzed as input factors: brightness, contrast, saturation, sharpness, and hue. In the context of using the image for clinical practice and at the same time performing a reliable sensitivity analysis, a careful selection of the ranges of variation of these factors is necessary. In the literature on deep dermatoscopy, typical choices for brightness and saturation are often set to multiplicative factors reaching a maximum of 1.5, that is, a 50% increase (Indraswari et al. 2022). For transformations that affect structure and focus, such as contrast and sharpness, the practice is often more conservative, limiting variations to a narrower range, typically 0.8–1.2, or a variation range of ±20% (Ratul et al. 2019). Hue is the most critical parameter. Variations are almost always kept within a very narrow range, limited to ±0.1, or a range of ±10% on a normalized scale, as greater alterations unrealistically distort the diagnostic color of the lesion (Indraswari et al. 2022). There is an inherent methodological trade‐off: increasing the range to the maximum possible (e.g., [0.0,2.0] for brightness, going from a completely darkened image to a completely illuminated one) exposes the model to variations that may not have any real clinical significance, introducing artifacts so extreme as to render the image non‐diagnostic. However, using minimal variations (e.g., ±5%) would render the Sobol index results meaningless, as the measured variance would not reflect the actual operational uncertainty. To address this trade‐off, we decided to establish uncertainty ranges by balancing the best practices of data augmentation in the literature with the need for a rigorous but clinically plausible robustness test. The chosen ranges are detailed in Table 1. These ranges are intended to represent “clinically plausible operational uncertainty,” encompassing typical variations that occur during image acquisition like differences in smartphone camera sensors, ambient light temperature, and variable distances between the lens and the skin surface.

**TABLE 1:** Parameter bounds for GSA.

Parameter	Range (bounds)	Variation
Brightness	[0.5,1.5]	±50%
Contrast	[0.8,1.2]	±20%
Sharpness	[0.8,1.2]	±20%
Saturation	[0.5,1.5]	±50%
Hue	[−0.1,0.1]	±10%

The VB‐SA assumes the independence among the photometric parameters considered. This assumption represents a modeling choice that enables variance decomposition and attribution of effects, rather than a claim about the true data‐generating process. In real clinical acquisition settings, some degree of correlation between photometric factors is expected. As a consequence, the reported sensitivity indices should be interpreted as first‐order indicators of risk drivers, potentially underestimating compound effects arising from correlated perturbations. Nevertheless, adopting independent factors allows for a transparent and reproducible sensitivity analysis, consistent with established practices in risk analysis, and provides a conservative baseline to identify dominant sources of diagnostic instability. Future work will explore the implementation of dependence‐aware sensitivity measures (see, e.g., Mara and Tarantola 2012) to explicitly account for correlations between imaging parameters.

The GSA was performed on a sample of 935 images from the hold‐out test set, with the objective of quantifying the contribution of the five defined optical factors to the total variance of the model's prediction output Y (the probability of melanoma). For the estimation of the Sobol index, the sampling was performed by generating N=256 base samples for the D=5 factors. This resulted in a total of N×(D+2)=256×7=1792 evaluations of the entire classification process for each analyzed image.

### 5.3 Computational Implementation and Reproducibility

The entire analysis was implemented using the Python programming language (version 3.11.13) on an Apple MacBook M2 with 8 GPU cores. For the classification architecture, we used TensorFlow‐metal/keras (version 1.2.0), while the GSA and the estimation of Sobol indices were performed using the SALib library (version 1.5.1), relying on its Quasi Monte Carlo sampling routines for variance decomposition.

To ensure the reproducibility of the GSA, we provide the technical details of the image perturbation pipeline. The framework was developed using the PIL.ImageEnhance module (v10.0) in Python. Perturbations were applied in a strict sequential order to minimize cumulative quantization errors and ensure consistency:

(1) Resizing to 128×128 pixels;
(2) Sequential application of brightness, contrast, sharpness, and saturation enhancements directly on uint8 data;
(3) Hue shifting in the Hue‐Saturation‐Value (HSV) domain.

The hue was manipulated by converting the Red‐Green‐Blue (RGB) image to the HSV color space. The shift ΔH∈[−0.1,0.1] was applied to the H‐channel as

(3) $$H_{\text{new}}=\left(H_{\text{old}}+\Delta H\cdot 255\right)\;\mathrm{mod}\;256,$$

where ΔH∈[−0.1,0.1] represents a normalized hue shift. The modulo operator ensures circular wrap‐around on the hue channel, preserving the cyclic structure of the HSV color representation.

Final perturbed images were cast to float32 before entering the model's internal normalization layer. Each enhancement step incorporates internal clipping to the [0,255] range and rounding to the nearest integer, effectively simulating the quantization noise and sensor limitations inherent in digital dermoscopy. This explicit specification of the perturbation pipeline ensures that the resulting Sobol indices can be correctly interpreted as variance‐attribution measures under the stated uncertainty model and implementation choices (Aloui et al. 2020).

Additionally, to ensure full computational reproducibility of the GSA, we provide the following technical specifications regarding the sampling design and the aggregation strategy:

- *Sampling design*: We employed Sobol quasi‐random sampling using the implementation provided in the SALib library (v1.5.1). The sampling procedure follows the Saltelli scheme for VB‐SA, which improves the efficiency of Sobol index estimation through structured low‐discrepancy sampling.
- *Deterministic seed*: To guarantee exact reproducibility of the sampling procedure, a fixed deterministic seed (seed=2021) was used in the Sobol sampler.
- *Computational load*: For each image, we used a base sample size N of 256. Given D=5 input parameters (brightness, contrast, sharpness, saturation, and hue), the total number of model evaluations per image was calculated according to the Saltelli sampling scheme as N×(D+2)=1792. For the entire test set of M=935 images, this resulted in a total of 1,675,520 independent model inferences.
- *Aggregation strategy*: Sensitivity indices (first‐order $S_{1}$ and total‐order $S_{T}$) were first computed individually for each of the M images to capture local variations in parameter importance. These results were then aggregated across the dataset by calculating the arithmetic mean and standard deviation to provide a global estimate of parameter influence:
(4) $$\bar{S}=\frac{1}{M}\sum_{i=1}^{M}S_{i},$$
  where $S_{i}$ represents the sensitivity index calculated for the ith image.

## 6 Results

GSA results are presented in Table 2. In the following, we use the notation $S_{1}$ and $S_{T}$ to denote the aggregated firt‐order and total‐order Sobol order indices, respectively, obtained by averaging the factor specific indices $S_{i}$ and $S_{T_{i}}$ across images. They quantify the impact of the five optical parameters considered on the variance of the DenseNet‐121 model's prediction output. At the aggregated level, hue and brightness emerge as the most influential parameters. Hue exhibits the highest total sensitivity index $S_{T}=0.524$, indicating that alone or in combination it contributes to more than half of the total variance. The brightness follows closely with an aggregated $S_{T}$ of 0.375. The saturation and contrast, with, respectively, $S_{T}=0.195$ and $S_{T}=0.110$, have a minor influence. Finally, sharpness is the least relevant factor, with a very low aggregate $S_{T}=0.022$, suggesting that alterations in sharpness have a minimal impact on model robustness. Analysis of the difference $S_{T}-S_{1}$ reveals the nature of the influence of each factor. For all parameters, the first‐order index $S_{1}$ is significantly high, indicating that the majority of the total variance is explained by the independent effect of the factor. Despite the dominance of direct effects, brightness and hue show the strongest interactions, with the difference $S_{T}-S_{1}$ reaching 0.150 in the cases of non‐melanoma for hue and 0.138 in the cases of non‐melanoma for brightness. This underscores that the general uncertainty attributed to these factors increases significantly when they act in combination. The indices can also be stratified for each diagnostic group. For the malignant class melanoma, the total sensitivity index is overwhelmingly dominated by the hue factor, with $S_{T}=0.573$. This suggests that color variation is the most critical source of uncertainty when evaluating a malignant lesion. For the benign class non‐melanoma, hue, and brightness, with, respectively, $S_{T}=0.512$ and 0.385 having strong index values. Notably, the $S_{T}$ for brightness here is close to that of hue, coupled with a high interaction contribution ($S_{T}-S_{1}=0.138$), indicating a slightly higher model sensitivity to luminosity changes when classifying benign lesions.

**TABLE 2:** Averages ± 95% confidence intervals (standard deviation in parentheses) of first‐order $S_{1}$ and total $S_{T}$ Sobol indices for the five optical parameters, aggregated across the test set and stratified by diagnostic class. The difference $S_{T}-S_{1}$ quantifies the contribution of interactions.

Optical	Aggregated indices		Indices by diagnostic category			
parameter	$S_{1}$	$S_{T}$	Category	$S_{1}$	$S_{T}$	$S_{T}-S_{1}$
Hue			Melanoma	0.451±0.123	0.573±0.105	0.122
	0.381±0.117	0.524±0.102		(0.231)	(0.219)	
	(0.226)	(0.215)	Non‐melanoma	0.362±0.115	0.512±0.102	0.150
				(0.221)	(0.213)	
Brightness			Melanoma	0.226±0.098	0.336±0.071	0.110
	0.242±0.103	0.375±0.080		(0.207)	(0.219)	
	(0.193)	(0.210)	Non‐melanoma	0.247±0.104	0.385±0.082	0.138
				(0.189)	(0.206)	
Saturation			Melanoma	0.097±0.066	0.168±0.042	0.071
	0.113±0.072	0.195±0.048		(0.090)	(0.109)	
	(0.106)	(0.122)	Non‐melanoma	0.117±0.073	0.202±0.050	0.085
				(0.109)	(0.125)	
Contrast			Melanoma	0.056±0.049	0.092±0.023	0.036
	0.065±0.054	0.110±0.028		(0.065)	(0.080)	
	(0.068)	(0.085)	Non‐melanoma	0.068±0.055	0.114±0.029	0.047
				(0.069)	(0.086)	
Sharpness			Melanoma	0.005±0.020	0.016±0.005	0.011
	0.009±0.023	0.022±0.007		(0.011)	(0.013)	
	(0.020)	(0.024)	Non‐melanoma	0.010±0.024	0.024±0.007	0.014
				(0.021)	(0.026)	

**FIGURE 4:**
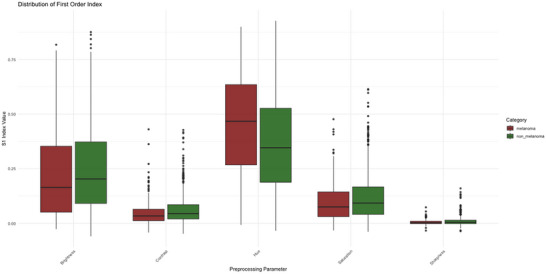
Distribution of first‐order Sobol index $S_{1}$.

**FIGURE 5:**
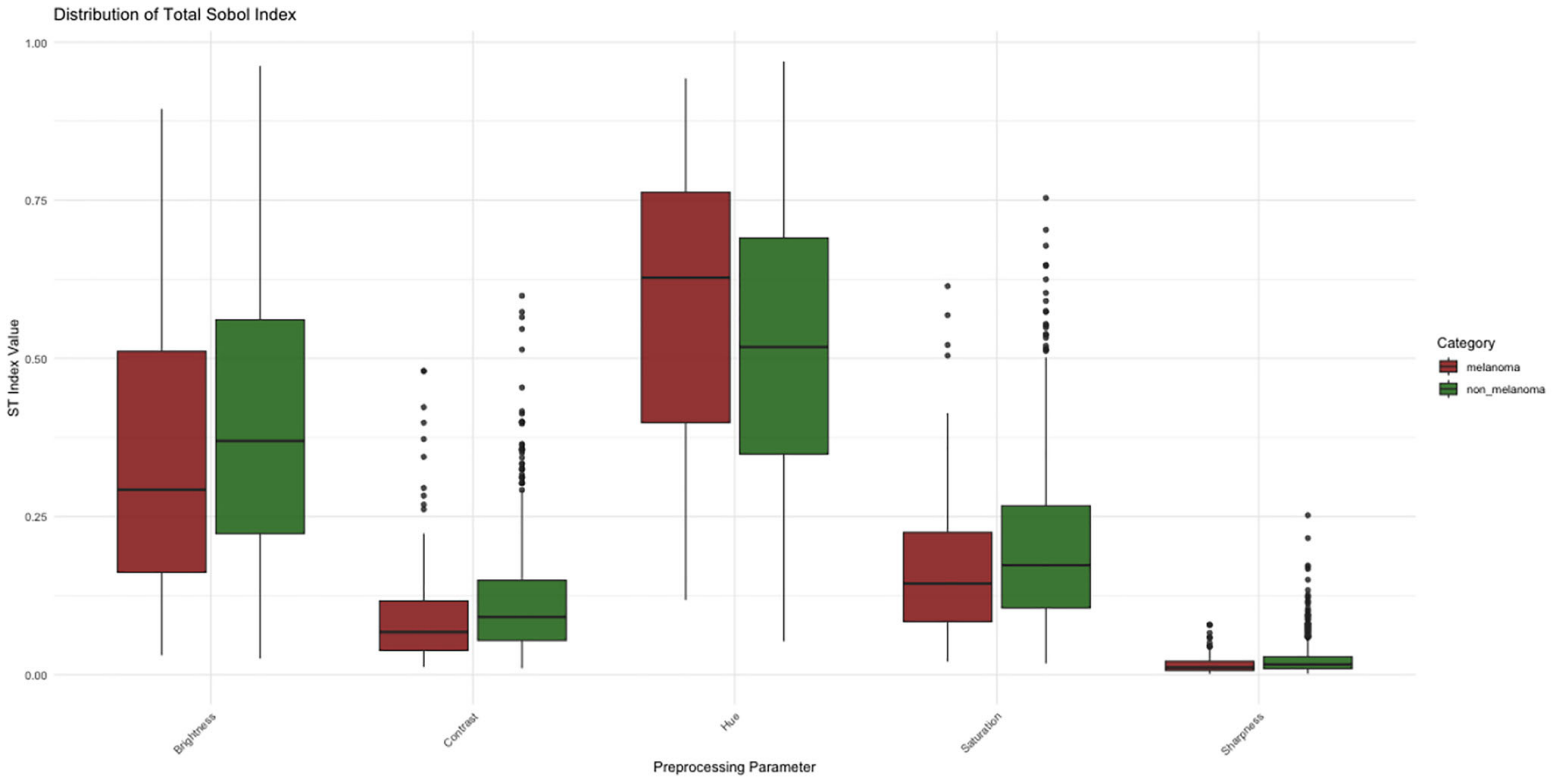
Distribution of total Sobol index $S_{T}$.

The results are also illustrated in the boxplots of Figures 4 and 5, showing the distribution of $S_{1}$ and $S_{T}$ across the replications. Hue and brightness display significantly greater dispersion and higher median values, confirming they are the primary drivers of uncertainty, consistent with the large standard deviations in Table 2. Sharpness and contrast show very narrow boxplots clustered near zero, indicating that their impact is consistently low across the dataset.

To assess the stability of the aggregated sensitivity indices when subjected to data reduction, we performed a stratified subsampling analysis of the test set, examining 20 independent subsamples within fractions ranging from 10% to 80%. The Sobol indices rankings derived from these subsamples were consistent across all fractions, thereby demonstrating the conclusions' considerable resilience to significant reductions in sample size.

To better understand the variance‐based sensitivity findings in the context of existing research on explainable artificial intelligence, we conducted an additional attribution analysis using Shapley values (Lundberg and Lee 2017). Shapley values offer a way to measure how much each input factor contributes to the model's output. They do this by averaging the factor's impact across all possible combinations of features, providing a theoretically sound way to assess feature importance.

Because the full CNN model is computationally expensive to evaluate within a Shapley framework, we estimated Shapley values using a surrogate model approach. Specifically, a Random Forest regressor was trained on the perturbation‐response dataset generated for the GSA analysis, where the five photometric parameters represent the inputs and the predicted melanoma probability is the target output. Shapley values were then computed using the TreeSHAP algorithm applied to the surrogate model.

The resulting feature importance values are reported in Table 3. The ranking obtained through Shapley attribution is highly consistent with the Sobol sensitivity analysis: Hue and brightness are the dominant contributors to prediction variability, followed by saturation and contrast, while sharpness remains negligible. This agreement between two independent attribution frameworks strengthens the interpretation that color‐related perturbations represent the main drivers of operational diagnostic uncertainty in the model.

**TABLE 3:** Mean absolute Shapley values (standard deviation in parentheses) for the five optical parameters, aggregated across the test set and stratified by diagnostic class.

Optical	Aggregated SHAP		SHAP by diagnostic category			
parameter	Mean		Category	Mean		
Hue			Melanoma	0.066		
	0.052			(0.034)		
	(0.034)		Non‐melanoma	0.049		
				(0.033)		
Brightness			Melanoma	0.041		
	0.036			(0.035)		
	(0.028)		Non‐melanoma	0.034		
				(0.026)		
Saturation			Melanoma	0.026		
	0.024			(0.015)		
	(0.015)		Non‐melanoma	0.023		
				(0.015)		
Contrast			Melanoma	0.016		
	0.015			(0.009)		
	(0.009)		Non‐melanoma	0.015		
				(0.009)		
Sharpness			Melanoma	0.004		
	0.004			(0.003)		
	(0.004)		Non‐melanoma	0.004		
				(0.004)		

The variance‐based GSA quantifies the impact of photometric perturbations on the continuous model output, that is, the predicted probability of melanoma. However, clinical decision‐making is ultimately based on threshold classifications. To complement the VB‐SA, we extended the robustness assessment to the stability of the predicted diagnostic label.

For each image in the test set, the probability output obtained from the original image was compared with the predictions generated under the 1792 photometric perturbations $p(\tilde{X})$. A classification threshold t=0.5 was used as a neutral decision boundary, as no clinically calibrated operating point was defined for the model. Binary labels were obtained by applying this threshold, and the resulting decision instability was quantified through the *flip rate*:

$$\mathrm{Pr}\left[1_{\left\{p(X)\geq t\right\}}\neq 1_{\left\{p\left(\tilde{X}\right)\geq t\right\}}\right],$$

which represents the probability that the predicted class changes under the perturbation distribution.

Across the entire test set, the aggregated flip rate was 0.142, indicating that approximately 14.2% of the perturbation scenarios lead to a change in the predicted diagnostic label relative to the baseline prediction. When stratified by diagnostic category, the flip rate was higher for melanoma cases (0.199) than for non‐melanoma cases (0.127), suggesting that malignant lesions tend to lie closer to the decision boundary and are therefore more sensitive to photometric perturbations, particularly when baseline probabilities are near the classification threshold.

To further characterize the dispersion of classification outcomes under perturbations, we computed the Gini index of the binary predictions for each image. In the binary case, the Gini index is proportional to the variance of the Bernoulli outcome and therefore provides a complementary measure of decision instability consistent with the variance‐based sensitivity framework. The aggregated Gini index across the dataset was 0.183 (SD =0.059), with comparable values between melanoma (0.166) and non‐melanoma (0.187) cases, indicating moderate variability in the classification outcomes under the perturbation distribution.

Finally, because the model output represents a probability, we evaluated its calibration using the Brier score on the test set. The overall Brier score was 0.176, with class‐stratified values of 0.290 for melanoma and 0.145 for non‐melanoma cases. This level of calibration indicates that the predicted probabilities maintain reasonable reliability, supporting the interpretation of the observed probability fluctuations as a consequence of operational uncertainty rather than purely numerical artifacts of the model.

These decision‐level robustness and calibration metrics are summarized in Table 4.

**TABLE 4:** Decision‐level robustness and calibration metrics under photometric perturbations. The flip rate quantifies the probability that the predicted class changes under the perturbation distribution. The Gini index summarizes the dispersion of binary predictions across perturbations, while the Brier score evaluates the calibration of predicted probabilities on the test set.

Category	Flip rate	Gini index	Brier score
Melanoma	0.199	0.166 (0.059)	0.290
Non‐melanoma	0.127	0.187 (0.058)	0.145
Overall	0.142	0.183 (0.059)	0.176

**FIGURE 6:**
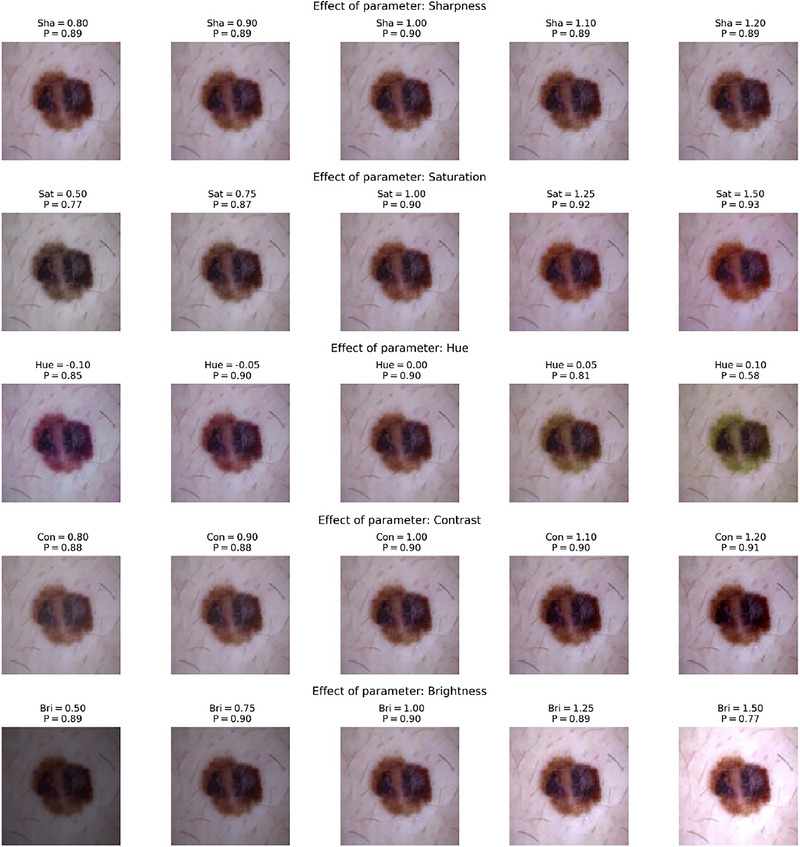
One‐at‐a‐time sensitivity analysis for a representative melanoma image. The predicted probability of melanoma P is shown as a function of individual photometric perturbations, while all other factors are held fixed at their nominal values.

**FIGURE 7:**
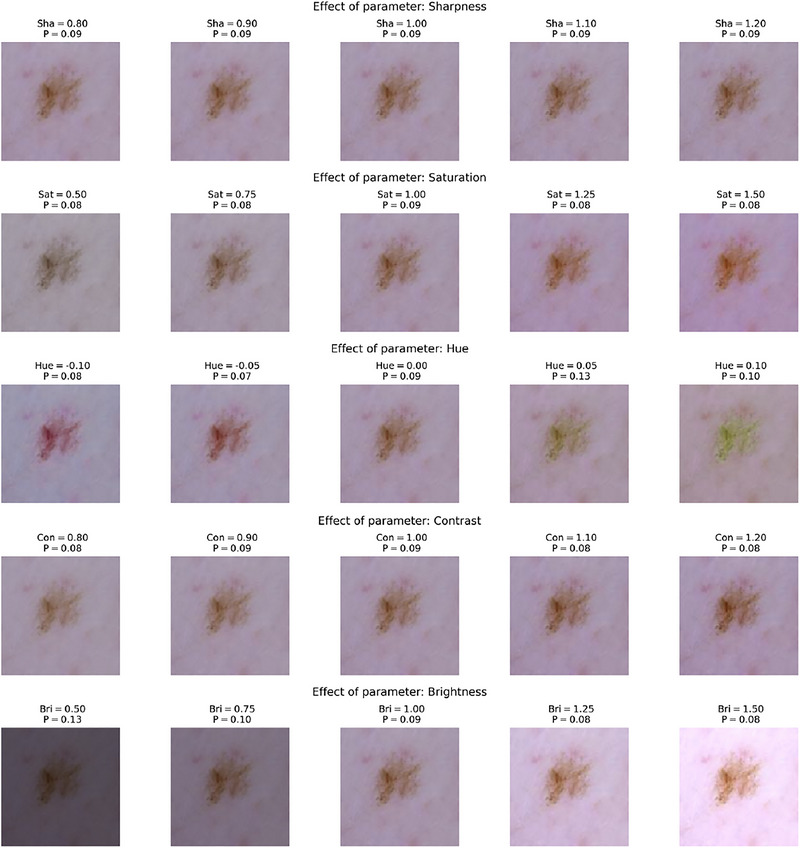
One‐at‐a‐time sensitivity analysis for a representative non‐melanoma image. The predicted probability of melanoma P is shown as a function of individual photometric perturbations, while all other factors are held fixed at their nominal values.

To visually show how the DenseNet‐121 model responds to controlled variations in individual optical parameters, we present in Figure 6 (an image with the true label *Melanoma*) and Figure 7 (an image with the true label *Non‐Melanoma*) a one‐at‐a‐time (OAT) sensitivity visualization.

We defined the ranges of variation for the five key optical parameters with the limits established for the GSA, while the other four factors are fixed to their respective neutral values (1.0 for scaling factors and 0.0 for shifts). The original image was then linearly sampled in n=5 steps for the parameter under investigation.

Seeing the output probability of the model P alongside these modified images, we can visually inspect the stability of the diagnostic prediction. For the image melanoma of Figure 6, in the hue parameter, we observed a dramatic shift in the confidence of the model: a hue shift of −0.10 resulted in a high‐confidence prediction of P=0.85, while a shift of +0.10 caused the probability to drop to P=0.58. This fluctuation in the output probability, given by a minor chromatic perturbation, visually confirms the high total Sobol indices $S_{T}$ and the model's chromatic fragility in critical diagnostic scenarios. For the sample with the true non‐melanoma label (Figure 7), the OAT visualizations often show more stable predictions with low probability.

However, the GSA reveals a more complex pattern. In fact, analyzing all variations and their interactions simultaneously, it becomes evident that even benign lesions are significantly sensitive to hue and brightness. This suggests that while a single factor might not always result in a misclassification, the joint variation of optical parameters represents a hidden vulnerability that can affect the model's robustness. Furthermore, although the model achieved a baseline AUC of 0.72 in the test set, the integration of GSA enriches the diagnostic evaluation. By prioritizing the most sensitive parameters during a targeted data augmentation process, future iterations can train the CNN to learn more invariant features, ultimately leading to both improved test metrics and higher clinical reliability.

**FIGURE 8:**
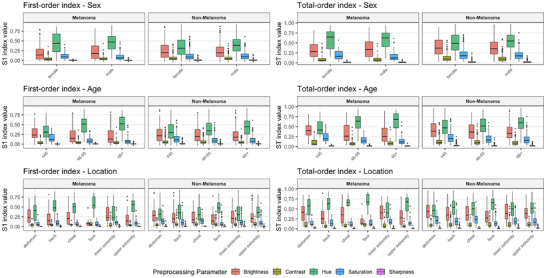
Subgroup sensitivity analysis stratified by diagnostic category for n=921 images of the test set. The left column displays the first‐order Sobol indices, while the right column shows the total‐order indices. The analysis is partitioned by biological sex (top), age groups (middle), and six most frequent anatomical localizations (bottom).

In Figure 8, we show the subgroup sensitivity analysis. After excluding cases with missing information, we obtained a complete metadata profile for 921 images, stratifying the Sobol indices across biological sex, age groups, and anatomical localizations. The results show that the dominance of hue and brightness remains remarkably stable across all diagnostic categories and clinical strata. This invariance suggests that the model's sensitivity is not conditioned by the clinical or demographic characteristics of the patient, but is a fundamental characteristic of the CNN's learned representations.

In summary, the GSA results consistently identify hue and brightness as the primary drivers of diagnostic uncertainty in the DenseNet‐121 model. In order to address the model stability and data dependency, we conducted additional robustness tests.

Finally, it is important to distinguish between operational sensitivity, the framework in this work, and structural sensitivity. Although the configuration of the model's hyperparameters influences the final weights of the CNN, the present study specifically aims to subject the model to post‐implementation clinical scenario testing. In this context, the model is treated as a “fixed” medical device, where the main source of risk stems from variability in image acquisition. Exploring how different training regimes or network architectures affect sensitivity profiles represents a distinct dimension of model robustness and constitutes a primary direction for our future research.

## 7 Conclusions

In this study, we explored the use of GSA to investigate how the optical parameters of an image impact the predictions of a DenseNet‐121 CNN model. The results show that the model's predictions are primarily determined by hue and brightness, which together account for most of the variance in the output. This high sensitivity to color and brightness parameters, often dominated by independent effects and significantly amplified by interactions, exposes a critical vulnerability in the diagnostic workflow. These results should be interpreted through the lens of “modeling ethics” (Saltelli et al. 2008). Sensitivity analysis reveals the impact of assumptions without necessarily justifying their defensibility. In our context, GSA acts as a “critical enemy” by revealing that model decisions are strongly influenced by factors such as slight variations in color or lighting. However, these are often artifacts of image acquisition rather than intrinsic biological characteristics of the lesion. This addresses what is described as the “illusion of uncertainty”: rather than examining irrelevant technical parameters, we focused on clinically plausible optical variations that represent operational uncertainties in the real‐world.

The implications of this work are threefold. For AI professionals, it provides a roadmap for targeted DA. NN users should take into account that the index ranking suggests prioritizing targeted data augmentation on hue and brightness, rather than applying generic or standard transformations when analyzing such images. In this way, the model can be trained to ignore acquisition noise and focus on morphological structures. For physicians, in the context of dermatoscopy, where the color of a lesion is a primary diagnostic indicator, our results offer a level of transparency. By quantifying how much a prediction might vary due to lighting or camera calibration, we provide the clinician with a reliability metric, helping them understand when a model's result might be influenced by suboptimal image quality rather than pathology. Another important insight we can draw from this analysis concerns the quality of the dataset. We could consider applying an automated pre‐filtering stage, which would automatically discard samples that are too unstable due to extreme values in hue and brightness that could invalidate the entire analysis. This would ensure that future models are trained on a robust dataset.

Therefore, this study goes beyond “useless arithmetic” by providing a transparent assessment of the robustness of the model. The predominance of hue and brightness in the sensitivity ranking suggests that future developments should prioritize color constancy algorithms. By identifying these critical sensitivities, we provide a clearer path toward the development of AI‐based diagnostic tools that are not only accurate in controlled environments but also stable in the face of diverse acquisition conditions typical of everyday dermatological practice. The proposed approach redefines, in some sense, the role of GSA in the context of XAI methods. While traditional XAI methods provide local, mechanistic explanations for individual images, Robust XAI via GSA shifts the focus to a global robustness diagnostic. In effect, it says something very powerful to the physician: *the model itself is an expert on image morphology, but remains chromatically fragile; it is an expert on shapes, but is confused by color changes*. In this way, GSA provides additional transparency information that goes beyond the individual prediction to explain the reliability of that prediction.

Since the current work focused on the binary classification of melanoma versus non‐melanoma to address the primary clinical objective, future work will focus on the natural extension of expanding the GSA to the full seven‐class spectrum of the HAM10000 dataset. This would allow for a more granular understanding of how optical perturbations affect the differential diagnosis between clinically similar lesions, such as distinguishing between pigmented benign keratoses and early‐stage melanomas, where color nuances are even more critical.

Moreover, we intend to refine the methodological framework in several directions. One possible extension is relaxing the assumption of parameter independence through the use of sensitivity indices specifically designed for dependent inputs. Another key future objective is the formalization of a sensitivity‐aware training framework. Rather than treating GSA as a post hoc evaluation tool, the results of the Sobol indices could be integrated directly into the training loop. Specifically, one can think of a GSA‐driven data augmentation: parameters identified with high total Sobol indices could be automatically prioritized in the Data Augmentation pipeline. By increasing the frequency and intensity of perturbations for the most sensitive factors, the model would be forced to learn more invariant and robust feature representations.

## Conflicts of Interest

The authors declare no conflicts of interest.
